# The effects of oral and topical corticosteroid in rabbit corneas

**DOI:** 10.1186/s12886-016-0339-5

**Published:** 2016-09-05

**Authors:** Kaoru Araki-Sasaki, Osamu Katsuta, Hidetoshi Mano, Takashi Nagano, Masatsugu Nakamura

**Affiliations:** 1 Department of Ophthalmology, Japan Community Health Care Organization, Hoshigaoka Medical Center, 4-8-1, Hoshigaoka, Hirakata city, Osaka 5738511 Japan; 2 Research and Development Division, Santen Pharmaceutical Co., Ltd., 8916-16, Takayama-cho, Ikoma city, Nara 6300101 Japan

**Keywords:** Corticosteroid, Keratitis, Cornea, Wound healing, Pharmacokinetics

## Abstract

**Background:**

To determine the most effective route of administration of corticosteroids in the treatment of ocular surface disease, by characterizing the difference between oral prednisolone and topical dexamethasone administration using an animal model.

**Methods:**

Pharmacokinetic analyses determined the corticosteroid concentrations in the normal ocular tissues of rabbits after oral or topical administration of corticosteroids using LC-MS/MS. In wound healing analyses, the area of the epithelial defect created by keratectomy using a 6-mm trephine was calculated with an image analyzer using an orally or topically steroid-administrated animal model. The average size of basal epithelial cells, the frequency of mitotic basal epithelial cells, the number of squamous cells, and the number of hypertrophic stromal fibroblasts were determined in the enucleated corneal tissues after wound closure.

**Results:**

By slit lamp examination, no remarkable differences were observed between orally and topically administered groups. Pharmacokinetic analyses showed that the distribution of dexamethasone after topical administration was superior to that after oral administration in the cornea. In contrast, both concentrations of corticosteroid applied topically and orally were similar with regards to AUCs (area under the concentration-time curve) in the conjunctiva. Although the healing rate was slower in the topical group, all corneas were almost healed within 96 h in the wound healing analysis. According to the histological analyses of epithelial cells, the average basal cell size was larger, the frequency of mitotic basal cells was greater, and the number of squamous epithelial cell layers was lower in the topically administered group although all of these differences were with no statistical significance. However, the number of hypertrophic stromal fibroblasts in the topically administered group was significantly lower than that in the orally administered group.

**Conclusions:**

There are different distributions and effects between orally and topically administered corticosteroids on the ocular surface. The data may provide the useful information in selecting the appropriate route of corticosteroid application for the treatment of ocular surface disease.

## Background

The administration of corticosteroids is an effective treatment for various kinds of ocular surface diseases, including treatment of allergic keratoconjunctivitis, autoimmune keratitis, recurrent corneal erosion due to dystrophy, acute hydrops of keratoconus, and endothelial dysfunction, although the use of corticosteroids in the treatment of infectious keratitis is still a controversial issue [1–6]. In such a condition of ocular surface, corticosteroids are administered by an oral or topical route. It is well-known that the bioavailability of oral corticosteroids is very high, with almost 100 % of the corticosteroid being absorbed. Although various kinds of oral corticosteroids are used, prednisolone is most often used in treatment of ocular surface, because of its moderate glucocorticoid effects, mild effects of electrolyte metabolism, and moderate half-life. On the other hand, topically applied corticosteroid will be expected to be high concentrations on ocular surface at the time of application and rapidly decrease by excretion of tears through the lacrimal duct. In our clinical experience, there were many patients who needed anti-inflammatory drugs in the treatment. However, there is no definitive evidence concerning which route of drug application is most suitable for each corneal disease. Therefore, it is important to obtain basic data about the differences between oral versus topical administration of corticosteroid.

The primary concern of topical steroid use for ocular surface diseases is delayed corneal epithelial wound healing [7, 8]. Occasionally, oral rather than topical corticosteroids are preferred in the case of corneal erosions to prevent delayed wound healing and secondary infection. However, the choice of oral or topical administration of steroids is often empirically based and not evidence based. In this report, we examined the differences in pharmacokinetics and wound healing of corticosteroids administered via either an oral or topical route using an animal model.

## Methods

Thirty-two male Japanese white rabbits (Kbl:JW; Kitayama Labes Co., Ltd., Nagano, Japan) weighing 2.6–3.2 kg were used in this study. All animals were treated and cared for in compliance with the Guiding Principles in the Care and Use of Animals. This study was approved and monitored by the Institutional Animal Care and Use Committee of Santen Pharmaceutical Co., Ltd., and all of the procedures were performed according to the Association for Research and Vision in Ophthalmology (ARVO) Statement for the Use of Animals in Ophthalmic and Vision Research.

### Pharmacokinetic analyses

Group 1 included 10 rabbits that orally received prednisolone (Sigma-Aldrich Japan, Tokyo, Japan), once a day for 3 days (0.25 mg/kg/day). Group 2 included 10 rabbits that received topical dexamethasone eye drops (0.1 % Santeson® ophthalmic solution; Santen Pharmaceutical Co., Ltd., Osaka, Japan) twice a day for 2 days, and once a day on the third day due to the euthanizing time schedule. On the third day, two rabbits from each group were euthanized at 0.5, 1, 2, 4, and 6 h after the final corticosteroid administration with an overdose of sodium pentobarbital (Somnopentyl®, Kyoritsu Seiyaku Co., Tokyo, Japan). After both eyes were enucleated, the cornea and conjunctiva were collected and stored at −80 °C until analysis (four eyes from two animals at each time point). The corticosteroid concentrations in the cornea and conjunctiva were determined using liquid chromatography/mass spectrometry (LC-MS/MS; Prominence/API4000; Shimadzu/Applied Biosystems, Kyoto, Japan).

### Wound healing analyses

Under general anesthesia and topical anesthesia, a round epithelial wound was created in the central cornea of the left eye of each rabbit by performing a 0.15-mm deep keratectomy with a 6-mm trephine. Based upon the approved experimental protocol, the right eye was used as the untreated control. The rabbits were randomly assigned to three experimental groups, with each group containing four rabbits.

Group 1 received prednisolone orally once a day for 5 days (0.25 mg/kg/day), Group 2 received 0.1 % topical dexamethasone eye drops twice a day for 5 days, and Group 3 (the control group) received saline eye drops twice a day for 5 days. All animals received antibiotic eye drops (1.5 % Cravit® ophthalmic solution, Santen Pharmaceutical Co., Ltd.) twice a day to prevent secondary infection.

All eyes were examined under a slit lamp biomicroscope (SL-15; Kowa Company, Ltd., Tokyo, Japan) at 0, 24, 48, 72, and 96 h after the wounding. The area of the epithelial defect was stained with fluorescein (Sigma–Aldrich) and photographed (Nikon Corporation, Tokyo, Japan). The total area of the defect was calculated with an image analyzer (WinRoof; Mitani Co., Tokyo, Japan). On the fifth day, the conjunctival injection and the stromal opacity were scored on a four point scale in a double blind manner (−, not remarkable; ±, slight; +, mild; ++, moderate; and +++, severe). One hour after the final corticosteroid administration on the fifth day, all rabbits were euthanized by exsanguination under deep anesthesia induced by a Somnopentyl® intravenous injection. Corneal specimens were obtained by enucleation and were histologically examined.

### Histological analyses

The excised tissues were immersed in F-G solution (9:1 of 10 % formaldehyde and 25 % glutaraldehyde) for 24 h and subsequently fixed with 10 % neutral-buffered formalin. Specimens were embedded in paraffin, cut into 4-μm thick sections, and mounted on slides. After deparaffinization, samples were stained with hematoxylin and eosin (H&E) and periodic acid-methenamine silver (PAM) to identify the basal membrane. The wounded area was observed under a scanning microscope (NanoZoomer; Hamamatsu Photonics, Shizuoka, Japan).

The area of the basal epithelial cell layer was measured with an image analyzer (HistoQuest; TissueGnostics, Vienna, Austria), and the number of basal epithelial cells was counted on PAM-stained slides. The average size of basal cells was calculated as dividing the basal cell layer areas by the number of basal cells. The frequency of mitotic basal epithelial cells was determined as dividing the total number of cells with a proliferated nucleus by the number of basal cells. The number of squamous cell layers was counted on H&E-stained slides, and that of hypertrophic stromal fibroblasts was counted and calculated as the number of cells per 1 mm^2^.

All values are expressed as the mean ± SEM. The data were analyzed using Tukey’s multiple comparison tests. A *P*-value of < 0.05 was considered statistically significant.

## Results

### Slit lamp examination

Table 1 shows the scores for the conjunctival injection and stromal opacity 96 h after the wound was created. Both scores were high in the saline group (Group 3). No remarkable differences were observed between orally and topically administered groups.

**Table 1:** Slit lamp examination at 96 hours after the wounding

	Group 1 (oral)				Group 2 (topical)				Group 3 (saline)			
Animal No.	1	2	3	4	9	10	11	12	13	14	15	16
Conjunctival injection	–	–	–	–	–	–	–	+	–	++	–	±
Stromal opacity	++	–	±	+	±	+	–	++	++	++	+	+

*No.* number

–: not remarkable, ±: slight, +: mild, ++: moderate, +++: severe

### Pharmacokinetic analyses

**Fig. 1:**
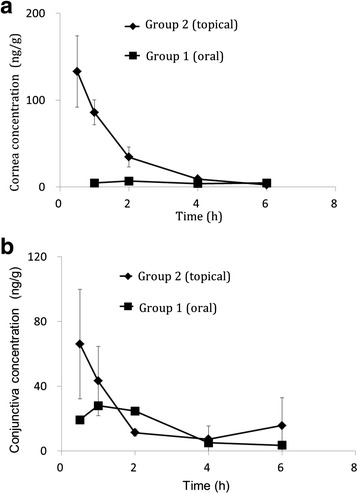
Corticosteroid concentration in the cornea and conjunctiva using oral and topical administration. Corticosteroid concentration in the cornea (**a**) and in the conjunctiva (**b**) using oral administration (Group 1) and topical administration (Group 2). Although corticosteroids administered orally did not sufficiently reach the cornea, this route maintained constant corticosteroid levels in the conjunctiva. The data are expressed as the mean ± SD (4 eyes from 2 animals at each time point)

The concentration-time curves of corticosteroids in ocular tissues are shown in Fig. 1. In the cornea, dexamethasone concentrations in the topically administered group (Group 2) showed a high C_max_ (133 ng/g) at 0.5 h after administration, and the area under the concentration-time curve (AUC_0–6 h_) was 204 ng · h/g. Prednisolone in the oral administered group (Group 1) was maintained at a low concentration throughout the observation period; C_max_ and AUC_0–6 h_ were 6.8 ng/g and 26.5 ng · h/g, respectively. However, in the conjunctiva of the oral administered group (Group 1), prednisolone concentrations were constantly maintained at 20–30 ng/g for 2 h after dosing. The dexamethasone concentration in the topically administered group (Group 2) increased (66.1 ng/g at 0.5 h) soon after the administration and immediately decreased at 2 h. AUC_0–6 h_ values in the conjunctiva for the orally administered group (prednisolone) and topically administered group (dexamethasone) were 81.3 and 113 ng · h/g, respectively.

Thus, in the cornea, the corticosteroid distribution after topical administration was superior to that after oral administration. However, in the conjunctiva, dexamethasone and prednisolone concentrations were similar, based upon the AUCs.

### Wound healing

**Fig. 2:**
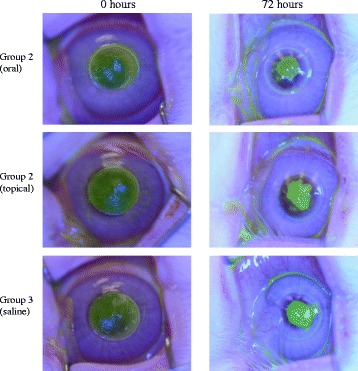
Wound healing by group. A representative case of wound healing by oral administration (Group 1), topical administration (Group 2), and by the control group (Group 3). Although the erosion present at 72 h appears to be greater in Group 2, this difference was not significant

**Fig. 3:**
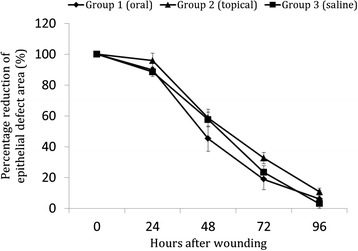
Wound healing as shown by the slope of the corneal erosion area. Although the wound healing was delayed in Group 2, erosion almost completely healed at 96 h. The data are expressed as the mean ± SEM of 4 eyes

Representative photographs from each group are shown in Fig. 2. The epithelial defect was slightly larger in the topically administered group (Group 2) than in the orally administered group throughout the observation periods. However, corneal erosions were almost completely healed within 96 h in all three groups. In Fig. 3, the wound healing process is shown as a change in the area of the epithelial defect. Four eyes still showed epithelial defects of 2.4 mm diameter at 96 h in group 2. The healing rate was slightly slower in the topically administered group (Group 2) than in the other two groups, but this difference was not significant.

### Histological analyses

**Fig. 4:**
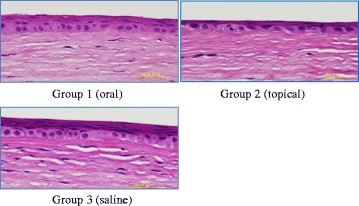
The flattened epithelium consisted of two layers in Group 2. Note the presence of three or more layers in Groups 1 and 3. Bar = 50 μm

**Fig. 5:**
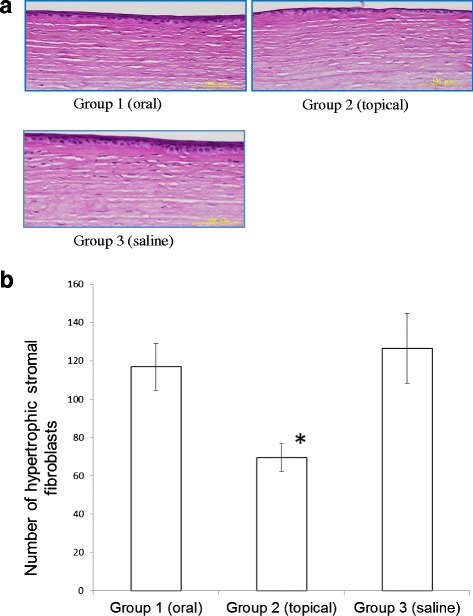
Stromal fibroblasts. The number of stromal fibroblasts was lower in Group 2 (topical steroid administration) as indicated by histological analyses (**a**, bar = 100 μm) and statistical significance [**b**, mean ± S.E.M, *n* = 4; *P < 0.05 versus Group 3 (saline)]

There were some differences in basal epithelial cells between the topically and orally administered groups by histological analysis (Fig. 4). The average basal cell size was largest in the topically administered group (Group 2) and smallest in the orally administered group (Group 1), although these differences were not significant (15.9 ± 1.7 μm^2^ in Group 1, 18.4 ± 1.7 μm^2^ in Group 2, and 17.4 ± 1.6 μm^2^ in Group 3). The frequency of mitotic basal cells was highest in the topically administered group (Group 2) and lowest in the orally administered group (Group 1) (1.0 ± 0.4 % in Group 1, 3.5 ± 1.8 % in Group 2, and 2.8 ± 1.1 % in Group 3). The number of squamous cell layers was lowest in the topically administered group (Group 2) with no statistical significance (3.0 ± 0.4 in Group 1, 2.3 ± 0.3 in Group 2, and 2.8 ± 0.3 in Group 3). The number of hypertrophic stromal fibroblasts was significantly lower in the topically administered group (Group 2) than in the other groups (116.8 ± 12.3 in Group 1, 69.5 ± 7.4 in Group 2, and 126.5 ± 18.2 in Group 3; Fig. 5a and b).

## Discussion

This is the first study that reports differences between oral and topical administration of corticosteroids on the ocular surface. Although there are many studies reporting differences between corticosteroid and nonsteroidal anti-inflammatory drugs on the ocular surface, there have been no studies reporting the differences based on the application route of the corticosteroids. In pharmacokinetic analyses, corticosteroids reach the cornea better when administered topically than orally, as was expected. High concentrations of corticosteroid were found just after topical application, and were soon followed by a rapid decreased in a few hours. This decrease will be more rapid in humans than in animal models because of the volume of tears. In contrast, only small amounts of corticosteroids by oral route reached the cornea. In conjunctiva, both of orally and topically administered corticosteroids were well distributed. Although the AUCs in conjunctiva were similar in both routes, oral administration maintained corticosteroid concentrations for a longer time period. Thus, corticosteroid through oral administration controls conjunctival inflammation better than topical administration and topical administration will be recommended to control the corneal inflammation. In the case of a wounded and diseased cornea, topical administration may provide a higher concentration than in a normal cornea, because of the lack of an intact epithelial barrier. Therefore, analyses of the pharmacokinetics in injured tissues should be investigated in future studies. In this experiment, we tried to remove various kinds of influences caused by the insults, and used only normal corneas. On slit lamp examination, we could not detect remarkable differences between corticosteroid and saline treatment regarding the grade of corneal opacity. However, less scarring after steroid application is often observed in clinical cases. This well-known phenomenon might be related to the differences in numbers of hypertrophic stromal fibroblasts observed in our studies. Thus, topical corticosteroid may be used if less scar tissue and opacity are required on the optic axis, and the corticosteroid should be applied orally if a reduction of scleral and ciliary inflammation is required.

We have applied 0.1 % dexamethasone eye drops twice a day as topical administration and 0.25 mg/kg prednisolone daily as an oral administration. These amounts of corticosteroid and the kinds of corticosteroids are based on our clinical doses.

The most controversial issue regarding topical corticosteroid use is delayed wound healing. Some previous studies reported a significant decrease in epithelial healing after steroid treatment [7, 8], while other studies reported the absence of such effects on corneal epithelial wound healing in humans and animals [9, 10]. We found that epithelial wound healing was delayed with topical corticosteroid administration, although this delay was not significant. The large basal cell size, high mitotic rate, and low squamous cell layers in group 2 indicated poor cell differentiation when treated with topical corticosteroid. However, a high mitotic rate in group 2 and complete healing of most corneas at 96 h even with topical corticosteroids (Group 2) suggest that topically administered corticosteroids do not always result in persistent corneal erosion. A longer time and larger scale examination will be required to confirm the conclusions regarding wound healing by corticosteroids. At present, our observations suggest that topical corticosteroids should be switched to an oral route when delayed epithelial healing is observed in clinical cases.

In choosing the route of steroid application in clinical practice, there may be other limitations such as intestinal absorption and movement disorders affecting the hands and the loss of visual acuity, which are essential to apply the eye drops. Usage of topical corticosteroid might also cause secondary infections on ocular surfaces. Although corticosteroids compromise the ocular surface, previous studies have shown that they do not affect the minimum inhibitory concentration of antibiotics or the proliferation of the organism [11–14].

In summary, we have shown different effects of corticosteroids on the ocular surface. These data may be useful in choosing the application route of anti-inflammatory treatments in ocular surface diseases. However, further larger scale investigations using various concentrations of different types of corticosteroids on injured or uninjured corneas should be conducted in future studies.

## Conclusions

This study showed the distributions and effects of orally and topically administered corticosteroids on the ocular surface. The data contributes to show the useful information in choosing the method of corticosteroid application for the treatment of ocular surface disease.

## Abbreviations

AUC: Area under the concentration-time curve
H&E: Hematoxylin and eosin
LC-MS/MS: Liquid chromatograph-tandem mass spectrometer
PAM: Periodic acid-methenamine silver
